# The Experiences of Carers of Adults With Intellectual Disabilities During the First COVID‐19 Lockdown Period

**DOI:** 10.1111/jppi.12382

**Published:** 2021-05-15

**Authors:** Varsha Patel, Gisela Perez‐Olivas, Biza Stenfert Kroese, Gemma Rogers, John Rose, Glynis Murphy, Vivien Cooper, Peter E Langdon, Steve Hiles, Clair Clifford, Paul Willner

**Affiliations:** ^1^ Birmingham Community Healthcare NHS Foundation Trust Unit 3 Priestley Wharf, Holt Street Birmingham B7 4BN UK; ^2^ Hertfordshire Partnership University NHS Foundation Trust 99 Waverley Road, St Albans Hertfordshire AL3 5TQ UK; ^3^ University of Birmingham, School of Psychology Edgbaston Birmingham B15 2TT UK; ^4^ Kent and Medway NHS and Social Care Partnership Trust, The Tarentfort Centre Bow Arrow Lane, Dartford Kent DA2 6PB UK; ^5^ Division of Psychiatry University College London Maple House, 149 Tottenham Court Road London W1T 7BN UK; ^6^ University of Kent Tizard Centre, Cornwallis North East Canterbury Kent CT2 7NF UK; ^7^ Challenging Behaviour Foundation, The Old Courthouse New Road Avenue, Chatham Kent ME4 6BE UK; ^8^ Centre for Educational Development, Appraisal and Research University of Warwick Coventry CV4 7AL UK; ^9^ Centre for Mental Health and Wellbeing Research, Warwick Medical School University of Warwick Coventry CV4 7AL UK; ^10^ Coventry and Warwickshire Partnership NHS Trust, Wayside House Wilsons Lane Coventry CV6 6NY UK; ^11^ Swansea Trials Unit, Clinical Research Facility, Institute of Life Science, Swansea University Swansea SA2 8PP UK

**Keywords:** carers, COVID‐19, intellectual disability, lockdown, parents

## Abstract

**Background:**

The recent COVID‐19 pandemic led to widespread international restrictions, severely impacting on health and social care services. For many individuals with an intellectual disability (ID) this meant reduced access to services and support for them and their carers.

**Aim:**

The aim of this study was to gain insight into the ways parents of adults with ID coped during the first 2020 lockdown period.

**Methods:**

Eight parents of adults with ID were interviewed. The recordings of these interviews were subjected to a thematic analysis.

**Results:**

Four main themes were identified: powerless and unappreciated; coping under lockdown; support; and the impact of lockdown on well‐being.

**Conclusions:**

The parents of adults with ID who made up our sample reported that they received little support from services and experienced a sense of powerlessness. Nevertheless, they were open to accepting support from family and friends and showed remarkable resilience. These findings are discussed in the light of the Willner et al. (2020) survey results on parental mental health and coping, and suggestions for future service provision during pandemic conditions are proposed.

## Introduction

The Coronavirus Act (2020) was implemented in an attempt to manage the first outbreak of the COVID‐19 pandemic in the United Kingdom and involved a variety of measures such as social distancing and social isolation for some. Within many mental health and disability services staff were redeployed to provide frontline work. For many adults with ID living with their families this meant less service provision (Redquest, Tint, Ries, & Lunsky, 2020). The charity Sense polled 1,000 people caring for a disabled adult family member in their household. The respondents reported taking on additional caring responsibilities during the first lockdown and noted an increase in challenging behavior of the disabled family member (Sense, 2020), in line with Alexander et al. (2020). They voiced feeling exhausted and let down by services during the first wave of the pandemic and had concerns about when support would be reinstated (Sense, 2020). Also, several news stories released during the spring of 2020 illustrate how the needs of informal carers of adults with autism were left unmet and how they had to deal with their offspring's frustration due to COVID‐19 restrictions and reduced support (BBC news, 2020). A qualitative study (Rogers et al., 2021) highlights how mothers caring for children with ID struggled with social restrictions and limited services during the first lockdown period, complementing other recent research on carers of children with ID (Asbury, Fox, Deniz, Code, & Toseeb, 2020; Toseeb, Asbury, Code, Fox, & Deniz, 2020).

The needs of informal carers supporting an adult family member with ID are likely to differ from those caring for children with ID as they rely predominantly on adult social care and health services and do not receive educational services (Evans, 2020). Willner et al. (2020) found that during the first COVID‐19 lockdown social restrictions and reduction in services meant a drastic change in the lives of adults with ID and their family carers and that due to the closure of adult day services and respite care, these carers had significantly less support than many carers of children, who, because of their child's special educational needs, had the option of letting them attend school.

People with ID have higher rates of comorbidities associated with poor outcome from COVID‐19 and have on‐going healthcare needs which require support during the pandemic (Kuper, Banks, Bright, Davey, & Shakespeare, 2020; Tromans et al., 2020). The loss of health and social services experienced during the first COVID‐19 lockdown period (Tromans et al., 2020), and the restrictions in place contributed to a decline in the mental well‐being of adults with disabilities, including those with ID, who reported feeling lonely and like a burden to others (Office for National Statistics, 2020a).

As the life span of people with ID continues to increase, aging parents may struggle with their own health issues impacting on their quality of life and those they care for (Chou, Lin, Chang, & Schalock, 2007). Older age is also associated with an increased risk of mortality in patients with COVID‐19 (Office for National Statistics, 2020b), placing older carers in the vulnerable category. Chou, Chiao, and Fu (2011) argued for interventions that create a supportive environment for carers of adults, especially those who are socially disadvantaged. They found that family carers of adults with profound and multiple disabilities had a lower health status, less formal social support and an inferior quality of life compared to carers of adults with less complex and multiple needs.

Any life change can add strain to the relationship between the carer and the adult they care for, even in “normal” times (Maggs & Laugharne, 1996). For example, qualitative research on the impact of life changes for people with ID and their carers found that the transition period from child to adult services can be especially stressful when families experience a sudden change and often a decrease in services (Neece, Kraemer, & Blacher, 2009). The restrictions implemented to manage the COVID‐19 pandemic during the spring of 2020 served to exacerbate such pre‐lockdown challenges, and there is emerging evidence that during the first lockdown the mental health of carers of children as well as of adults with ID was adversely affected over and above any pre‐exiting mental health conditions (Willner et al., 2020).

The full impact of the strict lockdown measures imposed to contain the first COVID‐19 wave on parents of adults with ID is as yet unknown. In “normal” times, better health in older parent‐carers of adults with ID appears to be linked to having a large and close support network of family, friends, and neighbors (Hill & Rose, 2009; Llewellyn, McConnell, Gething, Cant, & Kendig, 2010). These authors identified parents' main source of stress to be feeling helpless or not in control, together with poor professional support. Good access to information and resources, feeling included and accepted in the surrounding social environment and absence of financial barriers were found predictive of greater well‐being for parents caring for children and young adults with ID (Resch, Benz, & Elliott, 2012). It is therefore of concern that social support was least available to family carers of adults with ID with the most severe challenging behaviors and the least financial resources during the COVID‐19 pandemic (Willner et al., 2020).

As the pandemic unfolds it is becoming apparent that family carers of people with ID will continue to face challenges as public health restrictions continue. Their voices need to be heard to inform tailored care plans in time for future lockdown conditions. To this end, as part of a larger mixed method study (Willner et al., 2020) this qualitative study was designed to explore the recent experiences of lockdown conditions of the parents of adults with ID.

## Methods

### Ethics

Ethical approval was granted for the study by Swansea University Dept. of Psychology ethics committee (ref. 3874).

### Participants

Eight parents of adults with ID were recruited and interviewed. Their ages ranged from 44 to 82 years (mean = 66) with only two parents younger than 60 years.

The inclusion criteria for potential participants were as follows: (1) the main unpaid carer of an adult (18+) with ID, (2) access to the internet and comfortable with taking part in an interview, (3) living in the UK.

The age of the adult with ID ranged between 18 and 43 years (mean = 31). Further demographic details are presented in Table 1 below.

**TABLE 1:** Demographic information of parents and their adult sons/daughters with ID

Participant number	Gender of adult with intellectual disability	Degree of intellectual disability	Degree of autistic spectrum disorder	Degree of challenging behavior	Adult's age (years)	Relationship to the adult with intellectual disability
01	M	Moderate	Moderate	None	43	Mother
02	M	Severe	Severe	Moderate	18	Mother
03	M	Moderate	Severe	Moderate	32	Mother
04	M	Severe	Severe	Moderate	18	Mother
05	M	Severe	Mild	None	30	Father
06	M	Moderate	Not known	Not known	33	Mother
07	M	Moderate	Moderate	Moderate	30	Mother
08	F	Severe	Severe	Severe	43	Mother

### Procedure

Family carers of adults with ID living at home (*n* = 85) who completed an online survey presented via the RedCap online platform as part of a larger study (Willner et al., 2020) were invited to participate in an interview. From those who had expressed an interest in being interviewed (*n* = 55), 31 were selected at random by a member of the research team and invited to take part. Of these, eight parents responded and took part in the study. In addition to the consent already obtained for taking part in the main study (Willner et al., 2020) participants gave their verbal consent for this study after being read the information sheet by the researcher.

The first author (VP) conducted interviews by telephone (*n* = 7) or via video conferencing (*n* = 1) in June and early July 2020 (during or soon after strict national lockdown conditions) asking parents about their experiences during the first COVID‐19 lockdown period. Interviews were recorded and lasted between 19 and 40 min (mean = 31 min). Prior to interviewing, the schedule was practiced to ensure consistent delivery. Interviews were not conducted face to face due to the lockdown restrictions.

The interview schedule (Table 2), which was developed by the research team, that included a parent of an adult with ID, was identical to that used in our parallel study of parents of children with ID (Rogers et al., 2021).

**TABLE 2:** Interview schedule (prompts in italics were not asked if the participant had already addressed the topic)

(1) Can you tell me about what it is normally like to care for …? • What are the challenges? • What are the rewards? • What services do you normally receive (*school, day services, respite/short breaks*) • What support do you normally get? (*family, friends, etc*.)
(2) How have things changed for you since the COVID‐19 social isolation/distancing? • What things have stopped or changed (*services but also support from social networks*)? • How has daily life changed for you? • What have you found most challenging? (*Prompt for examples*) • How does it affect you? (*e.g., health, mood, sleep, routines, relationships*) • How does it affect the person you care for? • How does it affect the other people who live in your house? • Do you think your caring situation is understood by others?
(3) What support do you have at the moment? • Others in the household? • Professional support? • Friends and family? • Ask about types of contact, phone, email, Skype, Facebook, other social media, parent support groups, etc. • How do you get information—do you have someone to contact if you need help?
(4) Is there anything that you do not have at the moment that would help you manage better? • Ideas for other support? • Lessons learnt (*looking back …*)? • What are your main concerns right now?
(5) What helps you most to cope with the current crisis? • Do you have any tips for other carers? • Any changes that you will keep going when COVID‐19 social isolation/distancing finishes?

### Data Analysis

The Halcomb and Davidson (2006) method which does not require transcription of the interview recordings, was used for data analysis. This method has been argued to be a valid and cost‐effective means of data management, particularly suitable for mixed‐method investigations (Halcomb & Davidson, 2006). The advantages of using this method are listed in Table 3.

**TABLE 3:** Advantages of the Halcomb and Davidson (2006) method

The Halcomb and Davidson (2006) method was adopted as, according to the authors, it has the following advantages: The costs associated with transcription (time, physical, and human resources) are significant. The process of transcription is open to a range of human errors, including misinterpretation of content, cultural differences, and language errors. The use of field notes taken during an interview and immediately afterward has been found to be superior to the exclusive use of audio recordings that are subsequently verbatim transcribed. Field notes capture researchers' thoughts and interpretations during the process of listening to audio recordings. Audio recordings can be beneficial in assisting interviewers to fill in blank spaces in their field notes and check the relationship between the notes and the actual responses. This can reduce interviewer bias. Audio recordings allows supervisors certify that data reported by a researcher are true and accurate. Where there is ambiguity of meaning, the audio recording can clarify the intended meaning from the original source. Using the original recording of the conversation allows researchers to recreate the nuances of the conversation, such as voice, tone, and the specific language of participants, which may assist in more complex analysis.

Good inter‐rater reliability was established by means of another researcher (GR) analyzing one of the interviews and VP and GR discussing the themes identified. The final themes were discussed and agreed between four authors (VP, GP‐O, BSK, and GR) who are all experienced in conducting qualitative research.

## Results

The four main themes identified are presented in Table 4. Three of these themes (2, 3, and 4) consist of two subthemes.

**TABLE 4:** Super‐ordinate themes, subthemes, and participants contributing to each

Themes	Subthemes	Participants contributing
1/ Powerless and unappreciated		1, 2, 3, 4, 8
2/ Coping under lockdown	*a. I'm used to it …* *b. The importance of routine*	1, 2, 3, 4, 5, 6, 7, 8
3/ Support	*a. Lack of service support* *b. Technology*	1, 2, 3, 4, 5, 6, 7, 8
4/ The impact of lockdown on well‐being	*a. Son's/daughter's well being* *b. Own well‐being*	1, 2, 3, 4, 5, 6, 7, 8

### Theme 1: Powerless and Unappreciated

A number of parents reported to have felt powerless and not as much in control during lockdown as they were otherwise. One relatively young mother with an 18 year old son stressed that she needed to be seen to be in control for his and his siblings' sake.

“It's really quite scary because you just don't know, have I got the right things, how much food we've got, and of course the kids are looking at you to be strongly in control which is yeah, what I've always presented them with. As a parent, being in charge of everything was actually quite unnerving, because everything changed” (Participant 4).

There was concern among parents about the future and not knowing what was going to happen.

“Knowing what's going to happen, like having some sort of timeline about, you know, support, to be honest with you, you know, in terms of like, the day centres that he's due to start next month. Umm, they're sort of, hoping to open fairly soon, they're not a social services run organisation, you know, in the private sector, so they're going to open. I think there's been poor guidance on, umm, children and adults with learning disabilities” (Participant 2).

“Please don't go in lockdown again” (Participant 8).

The experience of lacking control was also said to have had a negative impact on their sons/daughters with ID.

“He just doesn't cope with grey areas at all. He needs definites [sic] and we can't give him a definite” (Participant 4).

Some expressed doubt as to whether other people were aware of and fully appreciated the challenges that they faced as parents of adults with ID during lockdown.

“They've got no clue, no clue at all, none. You've got … people that say, oh I know, if I had them for a week, they wouldn't be acting like that” (Participant 2).

### Theme 2: Coping Under Lockdown

In response to the question “What helps you most to cope with the current crisis?” two subthemes emerged; “*I'm used to it*” and *The importance of routine*.

Under this main theme participants reflect on their long‐term caring responsibilities for their adult child and how previous challenging situations have made them more resilient and confident. They also offer some “tips” to other caregivers.

#### 2a: “I'm used to it”

Some of the parents reported that many of their current caring responsibilities had already been routine before lockdown and that making sacrifices and putting their son/daughter first was what they had been used to.

“I am used to it, I mean there have been various times in our lives where we've had to have [son] because he's had his mental health crisis and umm, yeah I'm very resilient generally. I coped all the time when he was little” (Participant 3).

“We've been socially isolating for years, we've been doing this for years, … isn't hugely out of our norm, just the fact that everything else has stopped, that's the tricky bit” (Participant 2).

#### 2b: The importance of routine

Parents stressed the importance of a structured routine for their son's/daughter's well‐being.

“She's very dependent on her routine, she absolutely needs it” (Participant 8).

“I think obviously, umm, being consistent with [son] has helped and it has made me realise that he does need consistency” (Participant 3).

“Because of his attention deficit hyperactivity disorder, if he's not busy then that can lead to problems” (Participant 7).

### Theme 3: Support

This theme includes comments on and evaluations of the support available to these parents (3a) and the technology that had been useful for them during lockdown (3b).

#### 3a: Lack of service support

Parents reported that during the lockdown period they experienced a lack of support and communication from statutory services and many felt they had been left to cope alone.

“In terms of outside help, during this period, people like me are completely left on their own” (Participant 8).

“When the COVID‐19 pandemic began, our support all disappeared, we obviously couldn't go to the day service, which had been the way he would have got back to some sort of normality” (Participant 6).

On the other hand, some parents appreciated the support they received from informal sources such as family and friends.

“We've good support network of friends who are really good about playing games and chatting and things” (Participant 3).

“I've got a daughter who lives locally, so she does, she supports me with [son] and does some of his care” (Participant 7).

#### 3b: Technology

Many parents expressed their appreciation of support received with the aid of technology especially videoconferencing to keep in touch with their social network including family, friends, and day services:

“Had a family Zoom with eight people” (Participant 5).

“I mean, I have to say, they [video conferencing platform organised by day services] have been really great, without them I don't know what I would do” (Participant 6).

### Theme 4: The Impact of Lockdown on Well‐Being

The final theme describes the impact of lockdown on the cared for adults with ID (4a) as well as on the parents themselves (4b).

#### 4a: Son's/daughter's well‐being

Lockdown was said by parents to have had negative as well as positive effects on their son's/daughter's well‐being. Depending on the severity of their ID, the adults with ID had various levels of understanding of the meaning of terms such as COVID‐19 and the need for social isolation/distancing/shielding. A lack of understanding resulted for some in heightened anxiety.

“It's that constant balance between we don't want him to stress him out or we don't want him to worry or what he gets in his head. We don't know what he would think or how he would react really, but I mean he knows the word COVID‐19 but he doesn't, I mean we said that it's something that makes people poorly, but beyond that, he doesn't necessarily understand it. So we have to kind of say, you can't hug because of germs, or things like that, but he will still go in for a hug, and we're having to like, no you can't, you can't do that” (Participant 4).

“… [son] learnt quite quickly to stay away from people …” (Participant 5).

“I think actually, because I've been … consistent with him, umm … we've talked a lot more about his anxieties and I think he's probably come to face them more. Whereas before, I would have had him for a couple of days and then he'd be going away and doing whatever he does. Umm, and I think the consistency has probably been a positive. Umm … because normally, you know he just goes, I see him then after, six days later and by then things have got confused in his head again. So I think there has been a reward, in that, he has seemed to have finally got less anxious, so we've been able to cut down on his medication that he's been on” (Participant 3).

#### 4b: Own well‐being

Caring during lockdown was said to be relentless at times and parents described the impact on their physical as well as their mental health.

“I know that I've done my very best for him, but it's been hard and I know it's taken a toll on me” (Participant 7).

“You feel as though you're not always there” (Participant 3).

Participant 3 talked about her inability to practice mindfulness or enjoy her free time because even when her son was not present, she could not stop worrying about him.

Many parents mentioned exhaustion and a lack of time to relax.

“I've been exhausted, it's been busy … I don't have an awful lot of free time actually” (Participant 7).

“The relentless nature of caring when you're on your own with someone, it is relentless … It's physically buggered me, it's physically knackered me” (Participant 8).

Due to strict social isolation during lockdown parents reported feeling isolated and frustrated which sometimes resulted in family tensions.

“I think … caring can be incredibly isolating” (Participant 8).

“Not seeing people face‐to‐face and the isolation you feel” (Participant 3).

“We've had some family tension” (Participant 3).

In contrast to the exhaustion, frustration, and isolation some parents felt, some also spoke of positive aspects to lockdown which were considered to be beneficial for their own mental and physical health.

“Spoken to people more over this period of time, than I would normally because you're checking in on people to see how they are. We're keeping the community spirit. You know the neighbours I know loads of the neighbours now that I didn't really know. So it's been horrible, but I suppose there's been positives that have come out of it, in a weird way” (Participant 2).

“I'm getting out for a couple of walks; a nice long walk really helps” (Participant 8).

“It's the simple life that's a better life anyway” (Participant 2).

Some parents expressed gratitude and counted their blessings for the good things in their lives and the people and things that helped them cope.

“I'm quite lucky, I've got a husband who's, you know, we've been together a very long time, we get on. So you know we kind of, you know, we can bat things off against each other … We've got a nice house and we're lucky in that respect, so it's not a huge burden” (Participant 2).

“We've got so much to be thankful for; we all [family] get along really well … We live opposite a park. I try and look on the positives; I think I'm very lucky; I have two wonderful sons and a wonderful husband” (Participant 1).

“When you start spending time with her [daughter], you start to realise how much she can actually understand … I adore her; she's gorgeous” (Participant 8).

## Discussion

This study provides a unique insight into the perceived experiences of parents caring for an adult with ID during the first COVID‐19 lockdown period. A sense of powerlessness and a lack of control permeate their accounts. They found it difficult to give guidance or satisfactory explanations to their son/daughter for the drastic changes in day‐to‐day life, leaving both the adult with ID and their parents unnerved. This sense of powerlessness was also voiced by mothers caring for children with ID during the first lockdown (Rogers et al., 2021). Willner et al. (2020) found that a high level of defeat and entrapment (a concept closely related to hopelessness) observed in both parents of children and adults with ID during lockdown, was associated with heightened stress and an absence of social support (Willner et al., 2020). In “normal” times, feeling helpless and not being in control of a situation as well as poor professional support have all been identified as sources of stress for parents of adults with ID (Llewellyn et al., 2010). Parents in this study voiced their concerns about the future, wanting lockdown to be over and for it not to happen again. Similar concerns about future lockdown periods were reported in a survey of families caring for a person with ID (Sense, 2020).

The lack of support from statutory services reported by these parents is striking. Some mentioned feeling abandoned, which is consistent with findings of another recent study where family carers of children as well as adults with ID also reported on the loss of health and social services support during the first wave peak (Tromans et al., 2020). Mothers caring for a child with ID similarly reported feeling abandoned by professional services during the first lockdown restrictions (Rogers et al., 2021).

The majority of our parent participants caring for an adult with ID appeared to be accepting of, and grateful for, the support offered by family and friends. This is in contrast to (Rogers et al., 2021) where mothers caring for children with ID reported to feel reluctant to accept such support. A link, albeit weak, between mental health problems and receiving less support from family/friends was reported by (Willner et al., 2020) which may at least in part explain why older parents (who appeared more willing to accept support from family and friends) were less depressed compared to the younger parents.

Mothers caring for a child with ID during the first lockdown gave clear accounts of how their mental health had deteriorated with some reporting an increase in use of psychotropic medication (Rogers et al., 2021). The older parents in the current study talked about isolation and exhaustion but their narratives are less focused on their mental health and well‐being, which may be due to being part of an older generation which is possibly more reluctant to admit to, and openly talk about personal problems (Murray et al., 2006). There is also some evidence to suggest that the mental well‐being of older carers may be enhanced by being more accepting of their situation compared to their younger equivalents (Llewellyn et al., 2010). Moreover, Resch et al. (2012) have highlighted that parents of older children with ID may have developed more effective problem‐solving abilities and are better adjusted to their parenting role, also contributing to their well‐being.

Technology, especially videoconferencing was identified by the parents in our study as a useful source of support for keeping in touch with family and for maintaining links with day services and other parents of adults with ID. This is consistent with a review highlighting the usefulness of internet‐based support groups for carers of adults with ID as a way of keeping socially engaged (Perkins & LaMartin, 2012).

The accounts of the eight parents interviewed showed that they had found ways of coping and were relatively resilient despite the challenges posed by lockdown conditions. They stated that they were used to managing their caring responsibilities in accordance with pre‐COVID‐19 research, which found that parent‐carers of adults with ID have developed many self‐reliant coping strategies (Llewellyn et al., 2010; Resch et al., 2012).

Some parents in the current study emphasized how adults with ID benefit from consistency and keeping to a routine, corroborating other COVID‐19 research findings (Colizzi et al., 2020) and they described how their adult son/daughter with ID experienced anxiety due to their lack of understanding of COVID‐19 and of the lockdown measures imposed to control the pandemic, also reported by Tromans et al. (2020). A news story released in June 2020 illustrates how some people with ID were “terrified” by unclear COVID‐19 guidance (Mc Lean, 2020). Access to information and resources lead to greater well‐being in parents caring for children and adults with ID in “normal” times (Resch et al., 2012). Sadly, according to the parents we interviewed there was a lack of information and guidance that was accessible for adults with ID during the first COVID‐19 lockdown period.

Challenging behaviors were not often mentioned in our sample of parent of adults with ID, contrary to the findings observed in parents of children with ID (Rogers et al., 2021). A possible explanation could be the low and moderate levels of challenging behaviors of the adults with ID cared for in this study (see Table 1). Some of our parents' narratives suggest that adults with ID were relying more on their parents during lockdown than before the pandemic. Such dependence on family carers could be detrimental in the long term (Eley, Boyes, Young, & Hegney, 2009) and concerns about the first lockdown contributing to the loss of independence for adults with ID, who could previously rely on community services, have been raised (Evans, 2020).

Some of the parents caring for adults with ID voiced a sense of gratitude as they “counted their blessings” despite the challenges experienced during lockdown. The mothers of children with ID also reported embracing change and holding on to positives during the first strict lockdown (Rogers et al., 2021). These results corroborate that parenting journeys of those caring for a son or daughter with ID show both positive and negative aspects that can be experienced simultaneously (Dunn, Jahoda, & Kinnear, 2020; Resch et al., 2012).

The difficulties that parents caring for adults with ID experience in obtaining appropriate services is a long‐standing issue (e.g. Innes, McCabe, & Watchman, 2012) and older fathers have reported a sense of having to fight with the system, which contributes to their stress (Dunn et al., 2020). As the current COVID‐19 pandemic continues, statutory agencies should have an awareness of how battling to obtain adequate support and resources is taking its toll on older parents who are already more vulnerable to ill‐health (Dunn et al., 2020; Office for National Statistics, 2020b).

Our findings are based on a small, relatively affluent (Willner et al., 2020) and mostly female sample of parents of adults with ID. Research with socially and economically disadvantaged parents, who are often the most hard‐to‐reach as they may not have access to the internet, would provide valuable, additional information.

Further research is also needed on the role of the siblings (Eley et al., 2009) and partners in supporting parents of adults with ID. Moreover, the challenges that single parents may experience during the current pandemic (Willner et al., 2020) should be further investigated.

A rapidly growing body of literature highlights the impact of COVID‐19 on day‐to‐day life including communication and contact with others and access to support services for adults with ID (Embregts et al., 2020; Lake et al., 2021). These consequences of the pandemic are also reflected in the narratives of our sample of parents. In order to support families caring for an adult with ID, further research is required to devise effective, accessible and acceptable virtual and in‐person health and social care services for these families (Lake et al., 2021; Lunsky, Bobbette, Selick, & Jiwa, 2021), including staff training on how to enable service users to understand the virus and its effect on everyday life (Dean, 2020).

Parents spoke about the ways in which they coped during the lockdown period and provided “tips” for other parents. They mentioned that keeping to a routine and being consistent had contributed to their son/daughter's well‐being and that contact with neighbors and going for walks had been of benefit to their own well‐being.

Our findings have the potential to inform future care plans for adults with ID and their carers which, while considering adults' with ID need for autonomy and independence, may include personalized digital support (The King's Fund, 2020), neighborhood schemes and clear guidance in an accessible format for parents and the adults with ID they care for. Better communication about availability or changes to services is also needed and the effects of closure of day services on families requires more consideration. The current pandemic and the various and changing restrictions imposed on the population as a whole provides an ideal opportunity to rethink social care and design a system that is responsive to the needs of those families that include and care for an adult with ID.

## Conflict of Interest

The authors declare no potential conflict of interests.
